# Surgical and Functional Outcome after Resection of 57 Tentorial Meningiomas

**DOI:** 10.1038/s41598-019-51260-3

**Published:** 2019-10-10

**Authors:** Arthur Wagner, Ann-Kathrin Joerger, Nicole Lange, Bernhard Meyer, Ehab Shiban

**Affiliations:** 1 Department of Neurosurgery, Technical University Munich School of Medicine, Munich, Germany; 2 Department of Neurosurgery, Universitätsklinikum Augsburg, Augsburg, Germany

**Keywords:** CNS cancer, Disease-free survival

## Abstract

Tentorial meningiomas (TMs) may challenge the surgeon with their close association to neurovascular structures. We analyzed a consecutive series with regard to surgical and functional outcome following microsurgical resection. We retrospectively reviewed patient charts and imaging data of every patient with a TM resected at a single institution and compared surgical and functional outcomes between groups stratified by choice of approach. 57 consecutive patients from October 2006 to September 2017 were included, of which 75.4% were female; mean age was 60 years (range 31–90), follow-up data was available for 85.4% and reached a mean of 18.3 (range 2–119) months with a median of 14.5 months. 54.4% of TMs were located at the medial compartments of the tentorium, 45.6% at the lateral edges. Complete resection defined as Simpson grades I and II was achieved in 72% of all cases, without statistically significant differences for both subgroups (p = 0.532). 9 patients (15.8%) developed a new cranial nerve palsy postoperatively with the vestibulocochlear nerve affected exclusively in the lateral subgroup (8.8% of total), followed by disturbances of oculomotion (5.4%). After 12 months, 93.0% of patients with available follow-up after 12 months retained fully independent functional status without deficit. Despite providing a surgical challenge due to potentially complicated anatomical relations, the choice of an appropriate surgical strategy overall results in favourable oncological and functional outcome after resection of TMs.

## Introduction

Meningiomas affiliated with the cerebellar tentorium (tentorial meningiomas; TMs) represent 3–6% within the spectrum of posterior fossa meningiomas^1,2^. Even amongst this distinct classification, TMs may generate remarkably heterogenous surgical and functional outcomes depending on their specific location, with the primary treatment constituting microsurgical resection^3^. Extensive venous drainage and arterial supply surrounding the tentorium as well as the close vicinity of the cranial nerves make these lesions daunting and may necessitate an interdisciplinary strategy involving radiosurgery^4–6^. Characteristically, the operating surgeon is challenged to judge between striving for complete resection or preserving function and thus permitting regrowth. Over the course of the past two decades, some series were reported to detail surgical outcome, but evidence still remains scarce, owing to the low incidence of these lesions.

We therefore conducted a retrospective database analysis with emphasis on the functional impairment of associated neural structures in relation to the location of the resected TM on the tentorial plane.

## Patients & Methods

**Figure 1:**
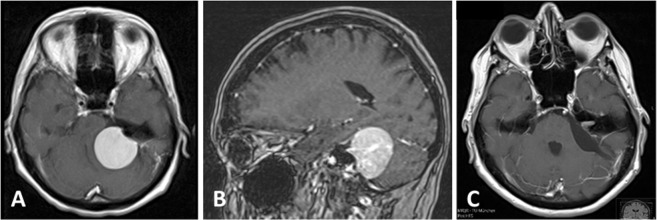
Left-sided *lateral* infratentorial meningioma in a 62-year-old female who presented with hearing loss and had confirmed impaired threshold of the middle frequency range on pure tone audiometry. Preoperative contrast-enhanced T1 MRI in axial (**A**) and sagittal (**B**) reconstructions. Postoperative imaging after resection (S2) via a left retrosigmoid craniotomy (**C**).

**Figure 2:**
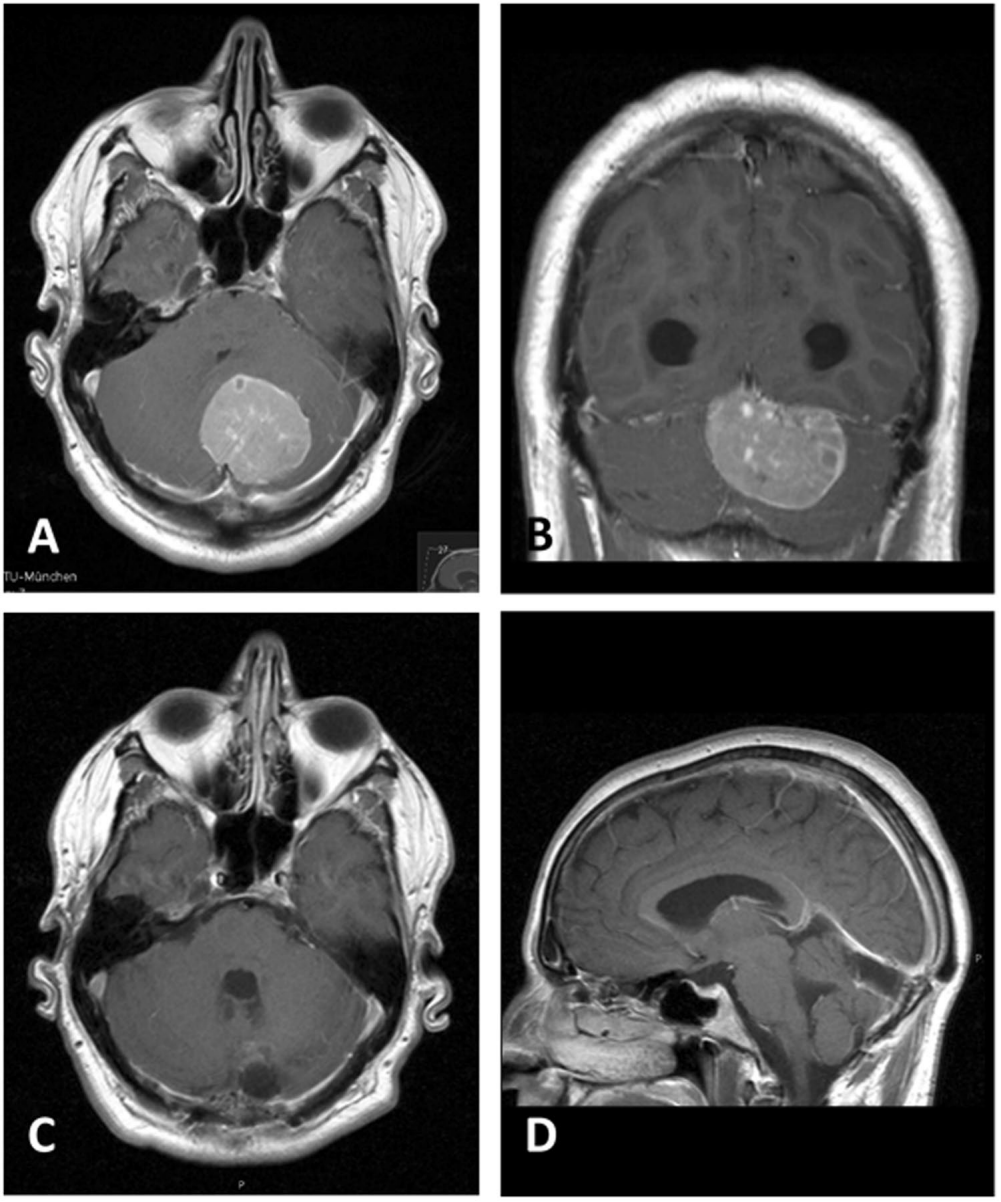
*Medial* infratentorial meningioma in a 43-year-old male who presented with accentuated headaches, nausea and vomiting. Preoperative contrast-enhanced T1 MRI in axial (**A**) and coronal (**B**) reconstructions. A transient gait ataxia was noted postoperatively. Imaging after resection (S2) via a median suboccipital craniotomy (**C**,**D**).

**Figure 3:**
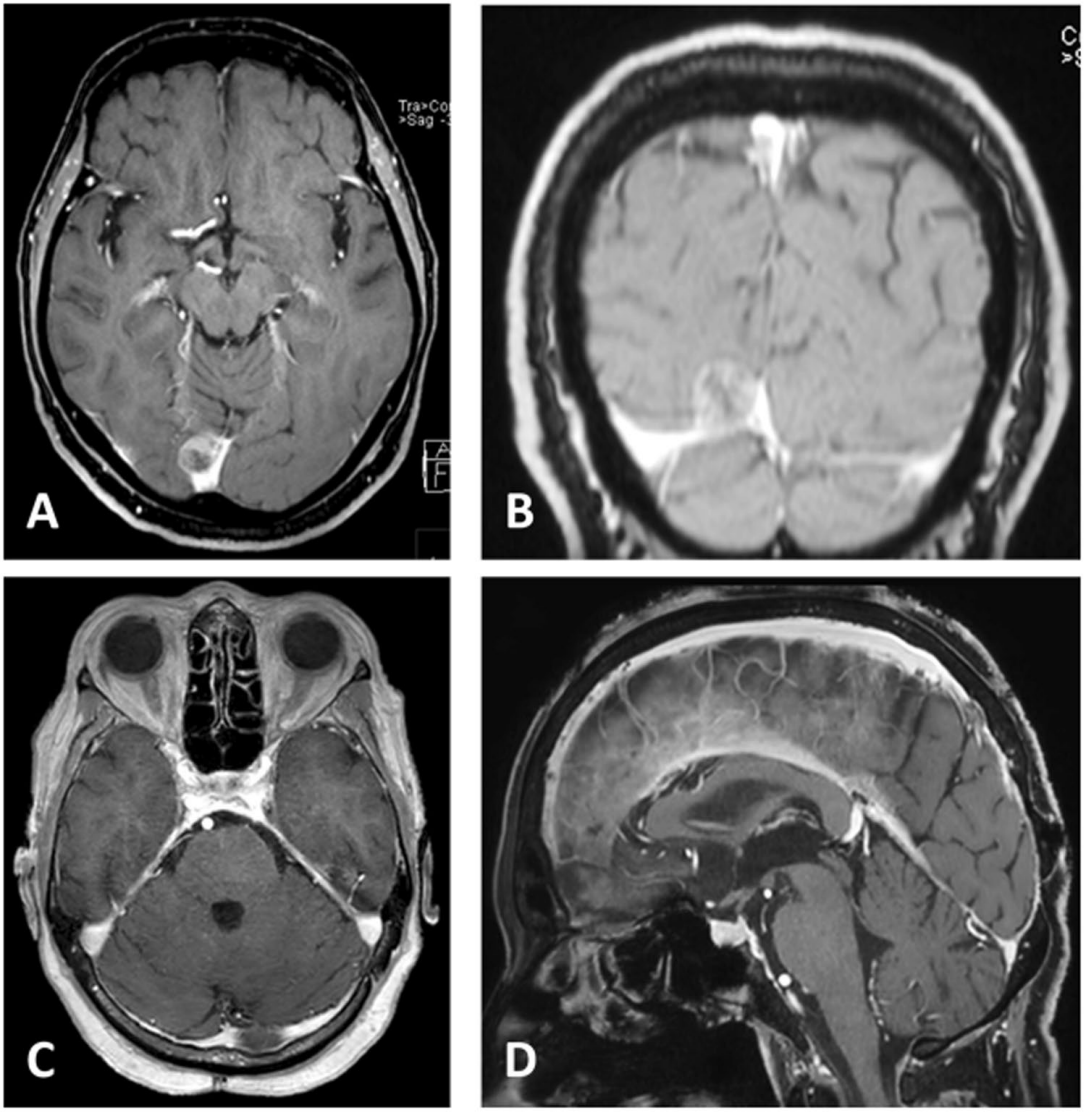
Incidental meningioma located in the right medial angle of the confluence in a 69-year-old female. Preoperative contrast-enhanced T1 MRI in axial (**A**) and coronal (**B**) reconstructions. The confluence was opened accidentally during resection of this rather small lesion that was intimately enclosed by the surrounding vessels. Imaging after resection (S2) via a midline parasagittal occipital craniotomy (**C**,**D**). The patient did not exhibit any deficit or complications postoperatively.

A retrospective review involved charts, imaging data and surgical reports of all patients with TMs resected at our institution. Meningiomas were stratified to those located at the lateral edges (*lateral* TM, Fig. 1) and those confined to the tentorial incisure, the torcular or the anterior edges of the tentorium in proximity to the cavernous sinus and cranial nerves (*medial* TM, Figs 2 and 3) as appraised on axial reconstructions of available imaging. The assigned category dictated surgical approach and technique. Typically, lateral infratentorial lesions were accessed via a retrosigmoid craniotomy in the supine position with the head rotated to the contralateral side by 90° and the ipsilateral shoulder supported on a soft pad. Median suboccipital and parasagittal occipital approaches were employed predominantly for TMs at the tentorial notch or TMs with additional supratentorial expansions that were situated too medially for a retrosigmoid approach. The patients were placed in a prone position and the head kept non-rotated, slightly inclined. Meningiomas situated anteriorly at the incisure required a subtemporal craniotomy, again supine with the head rotated 45–60° in the contralateral direction. The extent of resection was determined by the operating surgeon according to the Simpson grading scale^7^.

Preoperative status of cranial nerves, sensorimotor and coordinate functions was assessed through neurological examination. Loss of visual acuity and visual field defects were examined through ophthalmologic work-up including the standardized *Snellen* chart as well as *Goldmann* perimetry and reevaluated on follow-up. Contrast enhanced magnetic resonance imaging (MRI) represented the diagnostic imaging modality of choice, computed tomography (CT) was utilized as a complementary procedure and compensation for patients not eligible for MRI. Volumetry of preoperative and postoperative lesions was calculated with the *Brainlab Elements*™ software through accommodation of contrast-enhanced T1 sequenced in axial, coronal and sagittal reconstructions.

Postoperative imaging during the hospital stay was not routinely performed except for patients not harboring grade I lesions according to the World Health Organisation (WHO) grading or those that deteriorated with a new onset of neurological impairment, progressive headache or nausea. Otherwise, patients were routinely followed up with a cranial MRI scan and neurologically examined in our outpatient clinic after 3 months at the earliest.

For statistical analysis, IBM SPSS version 21.0 was used with Student’s t-Tests and Pearson chi-square testing applied for categorical comparisons^8^.

All procedures performed in studies involving human participants were in accordance with the ethical standards of the institutional and national research committee and with the 1964 Helsinki declaration and its later amendments or comparable ethical standards. The study group acquired approval by the local ethics committee, the requirement to obtain informed consent was waived (Ethikkommission der Technischen Universität München, Registration No. 5551/12).

### Ethical approval

All procedures performed in studies involving human participants were in accordance with the ethical standards of the institutional and/or national research committee and with the 1964 Helsinki declaration and its later amendments or comparable ethical standards. The study group acquired approval by the local ethics committee (Ethikkommission der Technischen Universität München), Registration No. 5551/12.

### Informed consent

For this retrospective study, the requirement for informed consent was waived by the local ethics committee.

### Disclosure

The authors declare that they have no conflict of interest affecting this study. The study was fully financed by the Department of Neurosurgery.

## Results

### Cohort & lesion characteristics

We analyzed consecutive 57 patients undergoing resection of a TM at our department between August 2006 and September 2017, 43 of which were female (75.4%). Median follow-up amounted to 14.5 months and mean follow-up was 18.3 months with a range between 2.0 and 118.8 months. Follow-up beyond discharge from hospital was unavailable in 14.6%. Baseline characteristics of the study cohort are displayed in Table 1. Forty-one (71.2%) TMs were located infratentorially, with an additional supratentorial expansion in three (5.3%) of these tumors. TMs were stratified to those confined to the tentorial incisure, the torcular or the anterior edges of the tentorium in proximity to the cavernous sinus and cranial nerves (*medial* TM; 54.4%) and those located at the lateral edges (*lateral* TM; 45.6%). A retrosigmoid approach to the affected side was used in 72% of all patients, of which one lesion. (1.8%) in this group had to be resected additionally through an occipital craniotomy in a second session.

**Table 1:** Baseline characteristics of study cohort.

	Total			Lateral	Medial P
Age at Surgery (years)		60 (31–90)	62 (31–90)	58 (37–81)	0.271
Preop. Size of Tumour (cm³)		3.1	2.9	3.3	0.359
Side	Left	47.4%	54.8%	38.5%	0.182
	Right	49.1%	45.2%	53.8%	
	Median	3.5%	—	7.7%	
WHO Grade	I	91.2%	90.3%	92.3%	0.390
	II	7.0%	9.7%	3.8%	
	III	1.8%	—	3.8%	

Preop. – Preoperative; cm³ - cubic centimeters.

The retrosigmoid approach was used exclusively for lateral TMs comprising the majority in our cohort (Fig. 1). A midline craniotomy was employed in 21.1% of all patients, of which 5 were suboccipital (8.8%) and 7 (12.3%) occipital (Fig. 2). For 3. (5.3%) TMs at the anterior margin of the incisure, the lesions were removed via a subtemporal approach.

Three patients (5.3%) were referred from another hospital with a recurrent TM, of which one had been treated by stereotactic fractionated radiation beforehand.

Table 2 lists the rate of topographical close relation of TMs to their neighboring structures as assessed on preoperative imaging. Osseous infiltration was uncommon (2 TMs; 3.5%). One TM infiltrated the posterior inferior cerebellar artery, another penetrated the cavernous sinus and affected the oculomotor and trigeminal nerves, resulting in diplopia and facial hypesthesia. No statistically significant difference concerning the association with neighboring cranial nerves or large veins was found between lateral and medial TMs (Table 2).

**Table 2:** Association of TM with cranial nerves and large sinuses on preoperative imaging, stratified by localisation.

	Lateral	Medial	P
Contact to Cranial Nerves	25.8%	15.4%	0.336
Contact to Sinus	41.9%	42.3%	0.977
Infiltration of Sinus with associated thrombus	16.1%	30.8%	0.189

### Preoperative complaints & functional status

Cephalgia and dizziness were the most common complaints precipitating the cranial imaging and detection of the TM in both subgroups, 84.2% of patients did not suffer from any signs or symptoms beyond the aforementioned and were in otherwise good health represented by a Karnofsky score of 100.

Five patients (9.0%) reported pronounced visual deficits preoperatively, two of these patients had mere perception of light and dark ipsilateral to a large TM that invaded the torcula and led to severe dislocation of surrounding structures with beginning hydrocephalus. No recovery of visual acuity was noted in any of these 5 patients after 12 months of follow-up. One patient with a right-sided median TM perceived a left-sided visual loss, which was not reproduced on follow-up examinations. There was no significant difference in the occurrence of visual degradation on presentation (p = 0.412) or postoperatively (p = 0.668) between subgroups of lateral and medial TMs.

Table 3 lists preoperative cranial nerve palsies other than visual impairment. The vestibulocochlear nerve was most commonly affected, especially in the lateral subgroup. Eight patients (14.0% of total) reported hearing loss to some extent on presentation, one of these had audiometrically confirmed surdity on their left ear corresponding with the site of the TM. No change was detected in any of these cases on follow-up and no difference between lateral and medial TMs concerning hearing loss was identified (p = 0.422).

**Table 3:** Preoperative rates of cranial nerve palsies, stratified between subgroups of lateral and medial TMs.

		Lateral	Medial	P
Preoperative Cranial Nerve Palsy	None	77.4%	88.5%	0.491
	III	—	3.8%	
	V	3.2%	3.8%	
	VI	3.2%	—	
	VIII	12.9%	3.8%	
	X	3.2%	—	

### Surgical outcome

The extents of resection (EOR) according to the Simpson^7^ grading system stratified by position on the tentorium are listed in Table 4, without statistically significant difference of EOR rates (p = 0.532). An ordinal regression model predicted higher Simpson grades for TMs that exhibited infiltrative growth into sinuses (p < 0.001; Fig. 3), but not for those in contact to cranial nerves (p = 0.807) as assessed on preoperative neuroimaging.

**Table 4:** Extent of resection according to Simpson grade, stratified by localisation.

		Lateral	Medial	Total	P
Extent of Resection	S1	29.0%	11.5%	21.1%	0.532
	S2	48.4%	53.8%	50.9%	
	S3	6.5%	11.5%	8.8%	
	S4	12.9%	15.4%	14.0%	

Three patients with TMs of WHO grades II and III received adjunct radiation therapy, in addition to 4 patients with extensive grade I tumor residuum that were deemed surgically unsuitable for another resection. Tumor recurrence was not detected in any case on follow-up.

One patient died 7 months postoperatively due to respiratory decompensation. This patient harbored a large median TM with supra- and infratentorial extension and invasion of the great vein of Galen that necessitated a two-staged procedure. The patient received adjunct radiation for residual tumor mass and deteriorated shortly after. No other fatalities were noted. Two patients (3.6%) had to undergo revision surgery for cerebrospinal fluid (CSF) fistula, after a retrosigmoid approach had been used in both cases. Three patients (9.7%) with lateral TMs and one patient (3.9%) with a medial TM underwent CSF diversion surgery for shunt-dependent hydrocephalus during hospital stay (p = 0.391).

Moreover, four patients (7.0%) suffered from new onset of cerebral venous thrombosis, of which three had medial TMs and 1 had a lateral TM (p = 0.221).

### Functional outcome

No patient was found to have a new sensorimotor deficit postoperatively.

In total, 9 patients (15.8%) who did not demonstrate any preoperative functional deficit developed new cranial nerve palsies during the immediate postoperative course, which most commonly included the vestibulocochlear nerve (8.8% of total; Table 5). Although the affection of VIII was exclusive to the lateral subgroup, no statistically significant difference was exhibited pertaining to the rate of new cranial nerve palsies overall (p = 0.125). By 12 months, hearing loss had significantly improved or fully resolved in all but two (3.6% of total) of these patients.

**Table 5:** Rates of new onset of cranial nerve palsies in comparison between groups in immediately postoperatively and on follow-up after 12 months.

	Cranial Nerve Palsy	Lateral	Medial	P
Postoperative	None	77.4%	92.3%	0.125
	III	3.2%	—	
	IV	3.2%	—	
	VI	—	3.8%	
	VIII	16.1%	—	
	IX	—	3.8%	
12 Months	None	90.3%	96.2%	0.289
	IV	6.5%	—	
	VI	—	3.8%	
	VIII	3.2%	—	

All but three patients (5.3% of total) retained impeccable visual acuity postoperatively, one patient with a large medial infratentorial TM experienced severe degradation of visual function to 0.05 in both eyes, which slightly recovered to 0.1 by 9 days postoperatively. No further recovery was noted in this patient after 6 months of follow-up. Another patient with a left-sided and laterally situated TM suffered from postoperative visual loss to 0.2 in the left eye, which had not recovered by 3 months. The third patient with a lateral TM that exerted a compressive effect on the mesencephalon and fourth ventricle reported only minor visual disturbance in one eye graded 0.8, but failed to return for follow-up examinations. Gait ataxia was noted in 13 patients overall (24.1%) without significant difference between subgroups (lateral vs. medial: 25.9% vs. 19.2%; p = 0.556). The majority of postoperative functional impairments (93.0% in patients with available follow-up) were transient and had fully resolved equally for both subgroups by 12 months (Table 5; p = 0.289). Both subgroups had a median Karnofsky score of 90 after 12 months.

## Discussion

In this publication, we retrospectively analyzed a consecutive cohort of 57 patients with a TM that were surgically treated at our institution. We aimed to describe the surgical and functional outcome with respect to tumor location and surgical approach. Various publications on skull base and posterior fossa meningiomas have contributed to the evidence in the recent decades and as is common strategy with any intracranial meningioma, an aggressive surgical management is advocated for optimal oncological outcome and progression-free survival^9^. Unsurprisingly, TMs represent a peculiar subgroup with difficult to manage hallmarks, most notably their location in between two compartments and close association to vital neurovascular structures. Brain stem compression and affection of cranial nerves as well as invasion into large draining vessels pose a challenge to the operating surgeon and are characteristic^6,10–12^. Overall, complication rates reached 23–34% in early series that applied a tailored approach for complete removal according to the lesion’s location on the tentorium, with CSF fistulae and hydrocephalus being the most common postoperative complications^9,13–15^. Subsequently, several series reported a subpar oncological outcome as a necessary trade-off for preservation of neurological function, although postoperative neurological morbidity still ranged between 23–33% in these analyses^12,16^. Reported mortality differed markedly between authors, Guidetti *et al*. reported a 9.8% fatality rate in 61 patients in 1988, whereas Gökalp *et al*. reached 2.7% in 1995^13,14^. Our own mortality rate seems comparably low with 1.8%, more so in contrast with early data stating surgical mortality of 29% by Barrows *et al*. and up to 44% by Frowein *et al*. in 1975^17,18^.

Complete resection classified as Simpson grades I and II was achieved in 72% of all patients in our series, with a trend towards subtotal resection in medially located TMs that was not statistically significant (Table 4) and TMs that invade sinuses (Table 2). These results are conform with those of recent publications^3,9,19,20^.

Postoperative cranial nerve dysfunction was seen occasionally in both laterally and medially located tumors, but transient in the majority of all cases by 12 months of available follow-up. New onset of vestibulocochlear dysfunction was exclusive to lateral TMs and mirrors the nature of the microsurgical intricacies incited by those lesions extending into the cerebello-pontine angle (CPA), corresponding to grades T6 and T7 of Yaşargil’s classification^21^. It occurs that there is a disparately more benign course of recovery for new cranial nerve palsies in comparison to those that had manifested before surgery, which generally do not rehabilitate on follow-up.

Some publications report explicit functional outcome: 9.7% of patients in a study by Colli *et al*. exhibited new cranial nerve dysfunction, specifically those with lesions at the inner tentorial ring^2^. Da Silva noted a rate of 32%, with 18% having persistent deficit on follow-up. In another publication by Bassiouni *et al*., 28% experienced cranial nerve dysfunction and 52% showed signs of gait ataxia, which coincides with our results. Bassiouni *et al*. meticulously detail the nature of the cranial nerve dysfunctions, with III, IV, V and VII being most commonly affected in the postoperative period, whilst the vestibulocochlear nerve was most commonly affected preoperatively^22^. In our study, vestibulocochlear dysfunction was predominantly found both preoperatively and postoperatively, which seems to correlate with the high rate of postoperative gait ataxia. Disturbances of oculomotion represented the second most common finding postsurgically, again transient in the majority of cases by last follow-up. The variability of functional deficit rates in the immediate course after resection of TM may be due to inconsistencies concerning the definition of a true manifest cranial nerve palsy, i.e. in cases of diplopia without oculomotor palsy during perimetry or tinnitus without objectifiable hearing loss through audiometry.

The classification and objectification of postoperative cranial nerve deficits will remain a quite difficile task on follow-up, which certainly may be ascribed to the heterogeneity of a patient’s self-perceived complaints, their fluctuating manifestation and their at times abstruse reporting to the surgeon. In our study, we emphasized a clear and distinct analysis of cranial nerve dysfunctions, which we saw rarely in the available literature. In addition, we stratified the intricate classification of the anatomical affiliation and origin of the TMs into two subgroups, hence depicting an immediate contrasting of functional deficits between the groups. The anatomical situation even within the very subgroup of TMs is notoriously diverse, as is abundantly evident in most investigations of the same kind, and may thus impede on any qualitative differentiation of surgical outcome between the many subtypes. With our proposed stratification into a succinct juxtaposition of two subgroups, the vulnerability of the 8^th^ cranial nerve with lateral lesions became accentuated and apparent.

The perception of anatomical associations between the tumor and associated surrounding structures greatly informed our choices of approach, aside from the obvious shortest distance to the cranial surface. The management of a large mass in the depths of a narrow surgical corridor may significantly endanger said structures when the approach neglects the projection of the surgeon’s field of view to behind the lesion and the possible displacement of vital surrounding structures. We therefore laid emphasis on assessing the preoperative imaging for the lesion’s contact to cranial nerves and vessels; albeit there was no statistically significant difference found, a clear trend between the subgroups was shown with regard to cranial nerve contact and associated sinus vein thrombosis. These again have been critical elements in establishing the preferred surgical route during the case series, allowing for clearer management of cranial nerves via a retrosigmoid approach into the CPA and midline approaches for lesions neighboring the sagittal and rectus sinuses, the torcular as well as the proximal transverse sinuses.

In essence, all of our results reflect the importance of an appropriate surgical strategy for a given TM. Moreso than with meningiomas in general, TMs require a surgical approach fit to the anatomical relation to neurovascular structures for optimal outcome. If such a paradigm is followed, those TMs in anatomically critical compartments share a comparable oncological and functional outcome to uncomplicated TMs.

### Study limitations

A most apparent limitation stems from the fact that this study discusses a retrospective cohort from a single center and should be clearly handled as such.

Further, albeit we are able to report a series without tumor recurrence, the validity of this assertion is severely hampered by the limited follow-up duration of our cohort and the follow-up duration, which renders a qualitative oncological assessment – of progression-free survival, for instance – difficult.

## Conclusion

Microsurgical resection yields favourable resection rates and functional outcome, remaining the treatment of choice for most tentorial meningiomas. Functional deficits are to be expected concerning auditory function in particular for lateral TMs, although these recover considerably during follow-up.

## Data Availability

The datasets generated during and/or analysed during the current study are available from the corresponding author on reasonable request.
